# Gut microbiota differences in five-year-old children that were born preterm with a history of necrotizing enterocolitis: A pilot trial

**DOI:** 10.1016/j.isci.2024.110325

**Published:** 2024-06-20

**Authors:** Amanda Magnusson, Seyedeh Marziyeh Jabbari Shiadeh, Maryam Ardalan, Diana Swolin-Eide, Anders Elfvin

**Affiliations:** 1 Department of Pediatrics, Institution of Clinical Sciences, Sahlgrenska Academy, University of Gothenburg, Gothenburg, Sweden; 2 Region Västra Götaland, Department of Pediatrics, The Queen Silvia Children’s Hospital, Sahlgrenska University Hospital, Gothenburg, Sweden; 3 Department of Physiology, Institute of Neuroscience and Physiology, Sahlgrenska Academy, University of Gothenburg, Gothenburg, Sweden; 4 Department of Clinical Medicine, Translational Neuropsychiatry Unit, Aarhus University, Aarhus, Denmark

**Keywords:** gastroenterology, microbiome

## Abstract

The study explores the long-term effects of necrotizing enterocolitis (NEC) on gut microbiota in preterm infants by analyzing stool samples from 5-year-old children using shotgun metagenomic sequencing. It compares children with a history of NEC, treated surgically or medically, to preterm controls without NEC. Findings reveal persistent gut microbiota dysbiosis in NEC children, with reduced species diversity and evenness, especially in those treated surgically. The surgical NEC group had a lower Shannon index, indicating less microbial diversity. Significant differences in taxonomic profiles were observed, mainly influenced by surgical treatment. These results underscore the lasting impact of NEC and its treatment on gut microbiota, suggesting a need for strategies addressing long-term dysbiosis.

## Highlights

• Gut microbiota dysbiosis after NEC may persist in preterm children until age five
• In preterm infants, surgery for NEC causes long-term gut microbiota dysbiosis
• *Akkermansia muciniphila* is the most common bacteria in the medically treated group
• *Escherichia coli* is the most common bacteria in the surgery-treated group

## Graphical abstract

**Figure:**
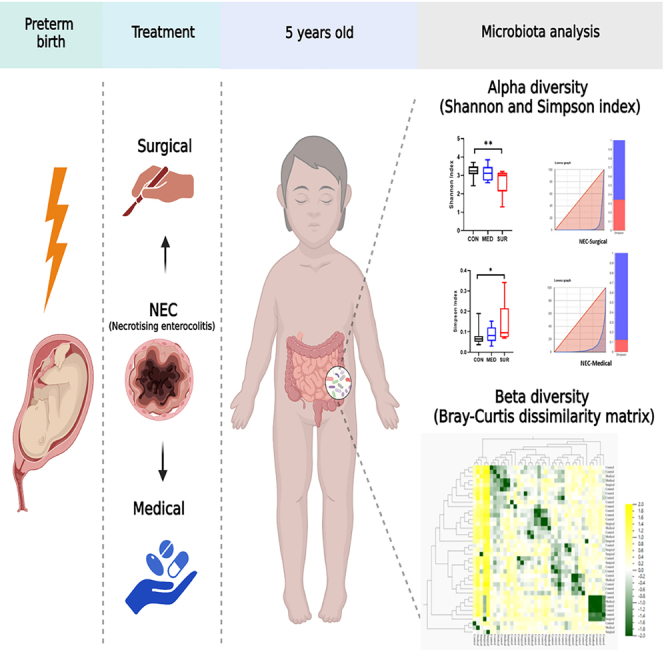

## Introduction

Necrotizing enterocolitis (NEC) is an acute intestinal inflammation caused by various factors such as prematurity, low birth weight, chorioamnionitis, and changes in blood vessel function, intestinal blood flow issues, and abnormal bacterial presence.^1^^,^^2^^,^^3^^,^^4^^,^^5^^,^^6^ The first option of NEC treatment is a medical approach. About 20–40% of preterm infants with NEC require surgery, and mortality rates remain high.^7^^,^^8^^,^^9^^,^^10^

A sequential process establishes the intestinal microbiota over the first years of life.^11^ Essentially, microbiota diversity increases with age, although it is still lower at five years compared to adult gut microbiota diversity. However, children’s gut microbiota undergoes significant changes during their first five years as their communities become more stable and adult-like.^12^^,^^13^^,^^14^^,^^15^ The homeostatic balance of the intestinal microflora is profoundly essential to the host, and a change in the microbial community can lead to gut vulnerability of pathogenic insult.^16^^,^^17^ Several factors are involved in the development of the microbiota, such as mode of delivery (vaginal or C-section), diet (formula or breast milk), and antibiotic consumption. Immaturity of the intestinal system, lack of bacterial diversity, and long-term antibiotic treatment increase the risk of disease.^18^^,^^19^^,^^20^ Preterm infants are more likely to develop early disease when they have abnormal microbial colonization. A recent study showed that preterm infant’s gut microbiota at four years of age was clustered with that of two-year-old, full-term infants, suggesting a delayed succession of preterm infant’s gut microbiota.^21^ Although NEC is a life-changing diagnosis, most current studies focus on the outcomes of patients during hospitalization up to the age of 2–3 years, focusing on poor growth and intestinal failure.^22^^,^^23^^,^^24^ An examination of the long-term complications of NEC showed that children with known or suspected NEC had an increased risk of functional impairment and bowel problems at seven years of age. There was no clear indication of how gut microbiota is affected, and whether NEC itself or NEC treatment are the primary factors in microbiota dysbiosis.^25^ Understanding the alteration of gut microbiota following NEC later in life is essential; the information potentially guides discussions about treatment options for patients with NEC and expands the understanding of how gut microbiota can be related to the long-term consequences of NEC. This is the first study that surveyed preterm NEC survivors until the age of 5 years regarding long-term gut microbiota and clinical characteristics by considering NEC therapeutic approach.

## Results

### Demographic and clinical characteristics of the NEC and control participants

**Figure 1:**
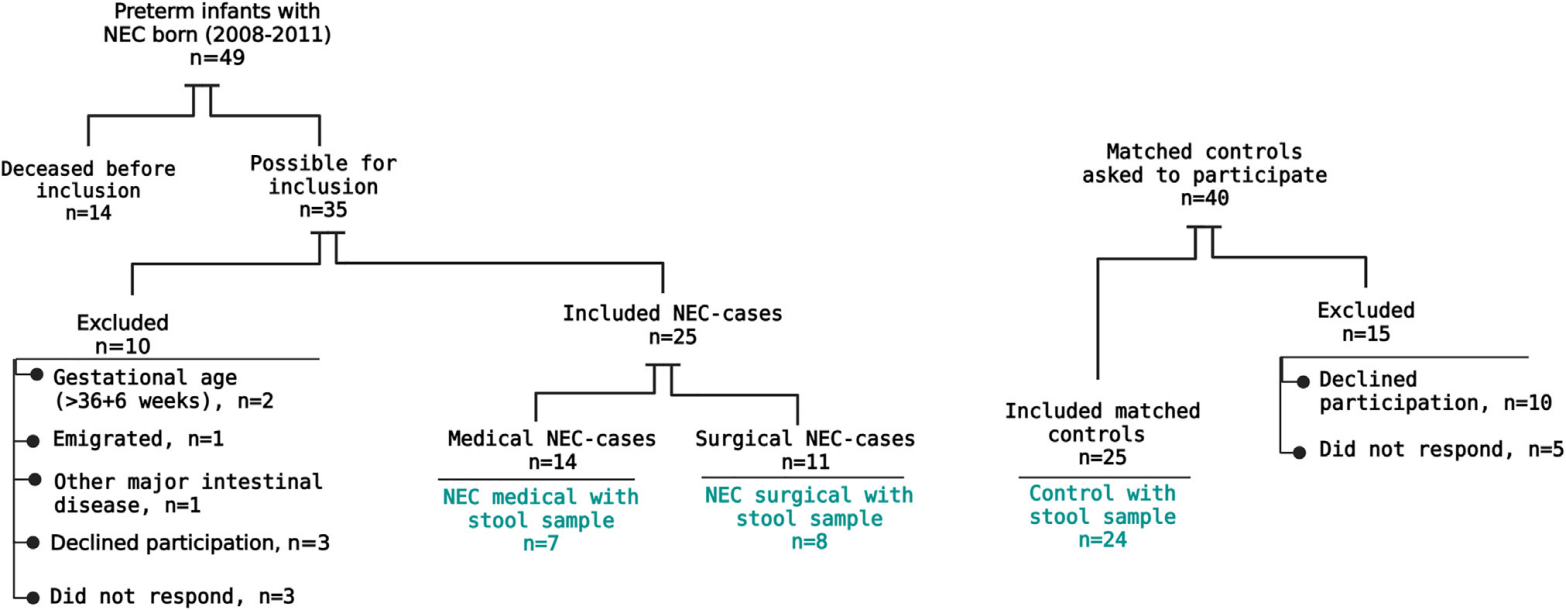
Flowchart depicting sample characterization and inclusion/exclusion criteria

Neonatal characteristics of the 15 NEC cases and the 24 controls are presented in Table 1. Seven (47%) of the 15 cases with NEC were treated medically for their NEC (NEC-medical), and 8 (53%) were treated with surgical intervention (NEC-surgical) (Figure 1). Details of the patient’s demographic characterization are found in the supplemental information. The NEC-surgical group was significantly shorter and had a significantly smaller head circumference at birth than controls. All infants with NEC and 21 of the 24 controls received antibiotics during their first admission to the neonatal intensive care unit (NICU). The median duration of antibiotic therapy at the first admission in the NICU among all infants with NEC was 31 days (14–89 days) (NEC-medical), and 36 days (23–89 days) among NEC-surgical. The corresponding median time for antibiotic therapy among the controls was ten days (range: 0–39 days). The median age when diagnosed with NEC was 13 days of age (range: 4–43 days). Our results indicated small effect size of the delivery type on the Simpson and Shannon diversity index respectively (Hedges’ g = 0.0, Hedges’ g = 0.4).

**Table 1:** Clinical characteristics of NEC-cases and controls

*Clinical characteristics*	*All NEC* n *= 15*	*Medical NEC* n *= 7*	*Surgical NEC* n *= 8*	*Controls* n *= 24*	*p value (All NEC* vs. *controls)*	*p value (Med NEC* vs. *controls)*	*p value (Surg NEC* vs. *controls)*
*Gestational age, weeks*	26 (24; 31)	28 (25; 31)	26 (24; 28)	27.5 (24; 35)	0.323	0.764	0.078
*Gestational age, days*	185 (168; 223)	196 (178; 223)	184.5 (168; 199)	194.5 (168; 246)	0.484	0.729	0.16
*Female sex, n (%)*	8 (53)	3 (43)	5 (63)	14 (58)	1	0.671	1
*Multiple gestation, n(%)*	6 (40)	3 (43)	3 (38)	9 (38)	1	1	1
*Cesarean delivery, n (%)*	9 (60)	3 (43)	6 (75)	11 (46)	0.514	1	0.229
*Birth weight, gram*	945 (470; 2320)	950 (645; 2320)	847.5 (470; 1130)	1032.5 (465; 2465)	0.191	0.872	0.07
*Birth length, cm*	35 (28; 44)	35 (32; 44)	32 (28; 39.1)	37 (29; 47)	0.138	0.800	0.046
*Head circumference, cm*	25 (20.4; 32)	26.3 (22.8; 32)	23 (20.4; 25.2)	25.5 (20.6; 32.5)	0.191	0.661	0.013
*PDA, surgical, n (%)*	3 (20)	1 (14)	2 (25)	3 (13)	0.658	1	0.578
*PDA, medical, n (%)*	7 (47)	1 (14)	6 (75)	10 (42)	1	0.372	0.22
*BPD*^a^*, n (%)*	6 (40)	1 (14)	5 (63)	12 (50)	0.745	0.191	0.412
*IVH grade 3–4*^b^*, n (%)*	1 (7)	0	1 (13)	2 (8)	1	1	1
*ROP stage 3-5*^b^*, n (%)*	4 (27)	1 (14)	3 (38)	5 (21)	1	1	0.393
*Abdominal surgery before 5 years of age*^c^*, n (%)*	12 (80)	4 (57)	8 (100)	1^d^ (4)	<0.001	0.005	<0.001
*Antibiotic treatment during first stay in NICU, n (%)*	15 (100)	7 (100)	8 (100)	21 (88)	0.271	0.335	0.555
*Days with antibiotic treatment during first stay in NICU, days*	31 (14; 89)	28 (14; 55)	36 (23; 89)	10 (0; 39)	<0.001	<0.001	<0.001
*Days with parenteral nutrition during first stay in NICU, days*	33 (13; 144)	28 (21; 51)	49 (13; 144)	9 (0; 21)	<0.001	<0.001	<0.001
*Age at sample collection, years*	5.1 (4.3; 6.0)	5.1 (4.3; 5.8)	5.1 (4.9; 6.0)	5.2 (4.5; 5.9)	0.831	1	1
*Height at five years of age, cm*	106.8 (98.7; 119.7)	107 (98.7; 119.7)	106.3 (102; 108.7)	108.7 (96.2; 117.9)	0.191	0.982	0.041
*Weight at five years of age, kg*	16.6 (13.2; 23)	17 (15.1; 23)	16.6 (13.2; 18)	18.4 (12.1; 22.9)	0.071	0.945	0.007
*BMI at five years of age, kg/m*^*2*^	14.86 (12.3; 16.2)	15.5 (14.1; 16.2)	14.6 (12.3; 15.7)	15.1 (13.0; 17.6)	0.502	0.695	0.174

Median (min; max) for continuous variables, number (%) for categorical variables. Mann-Whitney U test was used for continuous data. Fisher’s exact test was for categorical data. PDA, patent ductus arteriosus; BPD, bronchopulmonary dysplasia; IVH, intraventricular hemorrhage; ROP, retinopathy of prematurity.

a Value missing from one NEC-case.

b Values missing for one NEC-case and one control.

c 8 surgical NEC, 3 medical NEC with surgery later due to suspect post-NEC ileus and 1 medical NEC with resection of the gall bladder.

d Appendicitis at 2 years of age.

Four of the eight NEC-surgical had removed the ileocecal valve. Due to suspected post-NEC ileus, three of the seven NEC-medical required surgery on a later occasion, one of them with removal of the ileocecal valve. Overall, twelve (80%) of the infants with NEC required abdominal surgery at least once; eight NEC-surgical, three NEC-medical because of suspected post-NEC ileus and one NEC-medical with resection of the gall bladder and bile duct. One child in the control group had required abdominal surgery because of a ruptured appendix.

### Nutritional data during first admission

All 15 NEC cases had at least one episode of parenteral nutrition (PN), and all started PN during their first day of life. From the first day of life, all infants received at least minimal enteral feeding with human milk, either their own mother’s milk or pasteurized donor milk. The enteral feeding was increased as tolerated, primarily with own mother’s milk and donor milk secondarily. As a routine, none of the infants in the NICU received formula feeding before 35+0 weeks gestational age. When the amount of enteral feeding was between 70 and 100 mL/kg/day, a bovine-based fortifier was added to the human milk and hence, infants with an early onset of NEC developed the disease before the initiation of fortifier. No difference was seen between the NEC group and control group regarding the initiation of feeding.

During their first NICU admission, the median number of days on PN for control and NEC-surgical were 9 and 49 days, respectively. Our analysis revealed a positive correlation between the duration of PN and Simpson index and a negative correlation between PN and Shannon index in the NEC-surgical group (0.1 > *p* > 0.05) (Figure S1).

### Analysis of the microbiota taxonomic composition associated with NEC at 5 years of age: Sequencing results and taxonomic overview

On average, 32.9 M read pairs per sample were mapped to the gene catalog, representing 82.8% of the high-quality non-host (HQNH) reads (min = 78.2%, Table S1). Taxonomic profiling was performed using the MGS concept^26^ and the Clinical Microbiomics human gut MGS database.

### The relative abundance of microbial composition among control and NEC groups at the phylum level

**Figure 2:**
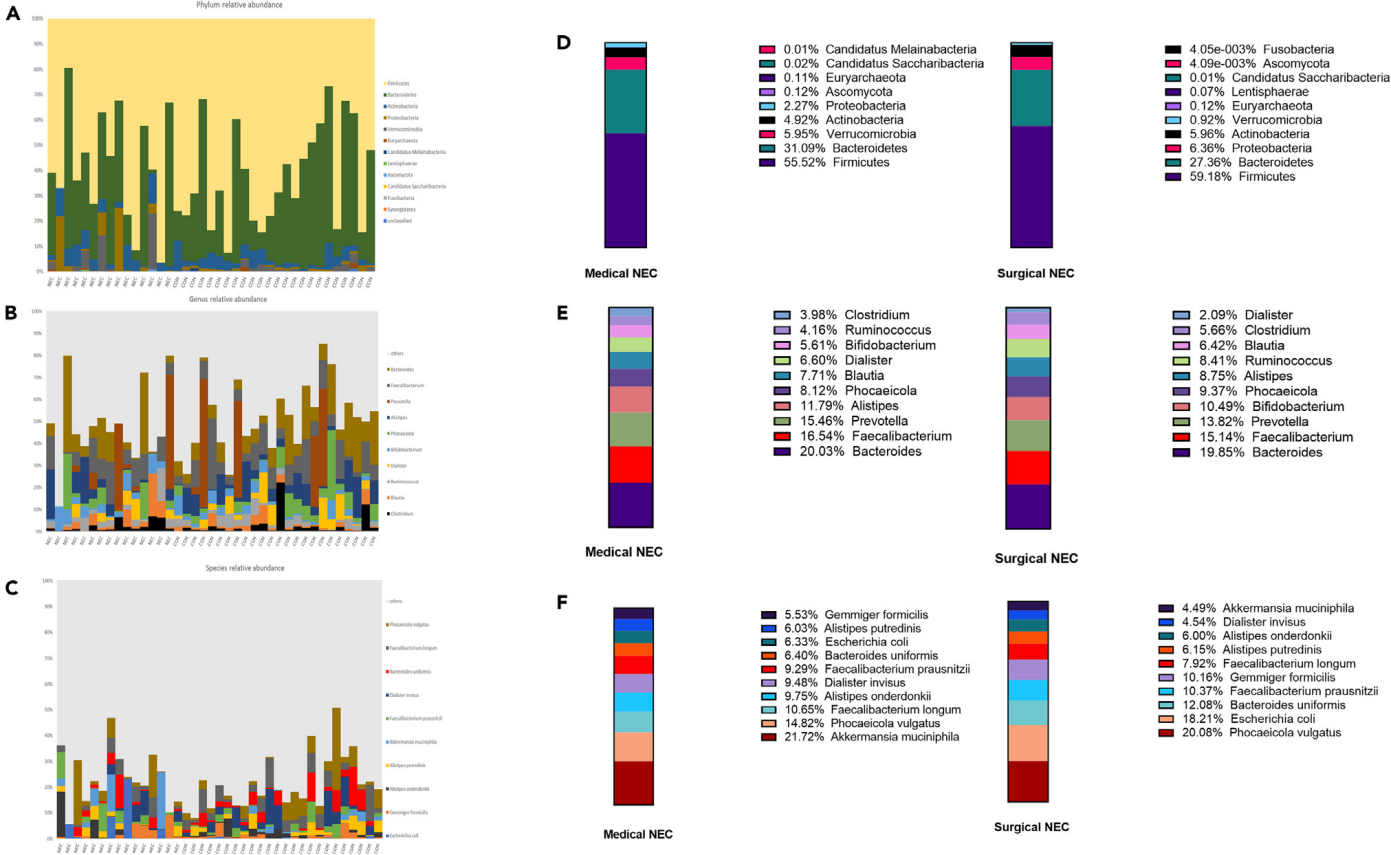
Taxonomic overviews of relative abundance of the top 12 taxa across all samples Taxonomic at the level of phylum (A), genus (B), and species (C). Light gray (others) indicates the total relative abundance of MGSs that are not in the top 10 most abundant taxa. Bar charts display the percentage of the most common bacteria at phylum (D), genus (E), and species (F) levels in NEC-medical and NEC-surgical treatment groups separately. See also Figure S2.

In this study, we tested the relative abundance of microbiota at different phylogenetic levels. Taxonomic profiles aggregated at phylum level are shown in Figures 2A and S2A).

### The relative abundance of microbial composition among control and NEC groups at the genus level

**Figure 3:**
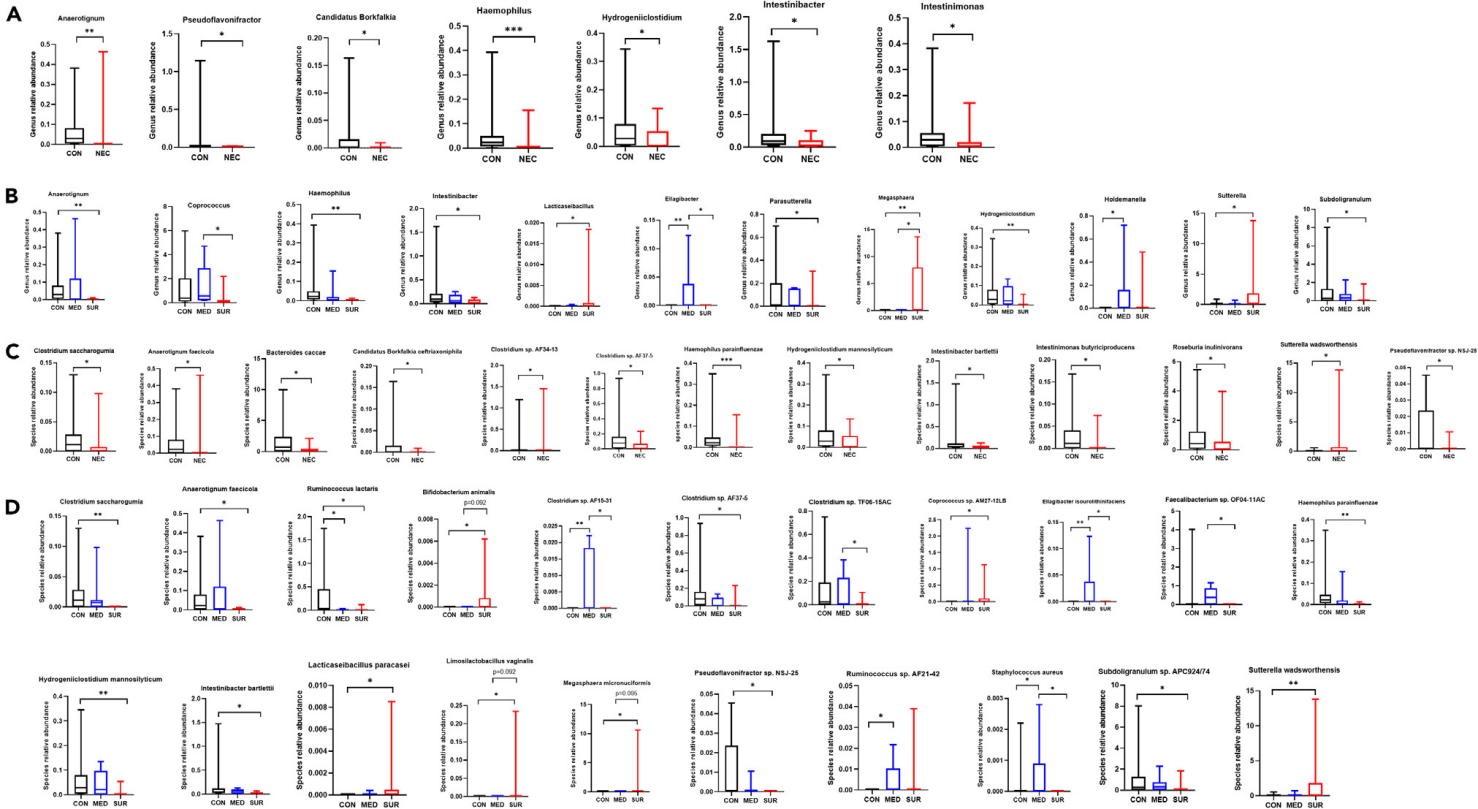
Relative abundance of taxa at genus level across groups Comparison of the relative abundances of taxa at the genus level between NEC and control participants (A) and between control, NEC-medical and NEC-surgical treatment groups (B). Comparison of the relative abundances of taxa at the species level in NEC and control participants (C). In addition, between control, NEC-medical, and NEC-surgical treatment groups (D). Data are presented as median, 1–99th percentile. Statistical comparison between control (*n* = 24) and NEC (*n* = 15) groups was performed using the non-parametric Mann-Whitney test and between the control (*n* = 24), NEC-medical (*n* = 7) and NEC-surgical (*n* = 8) groups was performed using the non-parametric and Kruskal-Wallis test. ∗*p* < 0.05; ∗∗*p* < 0.01; ∗∗∗*p* < 0.001. See also Figure S3.

Our results showed 167 bacteria; with 10 most common ones between the NEC-group and control-group presented in Figures 2B and S2B. There were remarkable differences in the relative abundance of *Haemophilus* (*p* < 0.001) and *Anaerotignum* (*p* < 0.01), which significantly decreased along with NEC. There were also significantly lower levels of *Candidatus Borkfalkia*, *Hydrogeniiclostidium*, *Intestinibacter*, *Intestinimonas*, and *Pseudoflavonifractor* in the NEC-group (*p* < 0.05) (Figure 3A). Despite not reaching statistical significance, there were trends toward an increase of *Sutterella* and a decrease of 5 other bacteria on genus level in the NEC-group compared to controls (0.1 > *p* > 0.05) (Figure S3A).

### The relative abundance of microbial composition among control and NEC groups at the species level

In terms of bacterial species relative abundance, 392 species were detected. The top 10 taxa with the highest average abundance across all samples are shown in Figures 2C and S2C.

*Haemophilus parainfluenzae* was the bacteria that reached the strongest statistical significance with low abundance in the NEC-group (*p* < 0.001). Additionally, there was significantly increased relative abundance of *Clostridium* sp. *AF34-13* and *Sutterella wadsworthensis* in the NEC-group, whereas the relative abundance of *Intestinibacter bartlettii*, *Intestinimonas butyriciproducens*, and *Roseburia inulinivorans*, among others, were significantly decreased in the NEC-group (*p* < 0.05) (Figure 3C).

Moreover, *Coprococcus eutactus*, *Faecalibacterium longum*, and *Roseburia hominis*, among others, showed a general trend of decrease in the NEC-group (0.1 > *p* > 0.05) (Figure S3C).

### Taxonomic composition in treated (medical/surgical) NEC and controls at 5 years of age

#### The relative abundance of microbial composition in treated (medical/surgical) NEC and control at the phylum level

We observed different bacteria with different percentages in each NEC case with surgical or medical treatment; the third most common phylum in the surgical group was Proteobacteria, while the third most common phylum in the medical group was Verrucomicrobia (Figure 2D).

#### The relative abundance of microbial composition in treated (medical/surgical) NEC and control at the genus level

The 3 most common bacteria were identical in the NEC-surgical and medically treated NEC cases*,* but the proportion was slightly different between the two groups (Figure 2E).

There were significant decreases in the relative abundances of *Anaerotignum*, *Haemophilus*, and *Hydrogeniiclostidium* in the NEC-surgical group (*p* < 0.01) and significant increase in the relative abundance of *Ellagibacter* in the NEC-medical group (*p* < 0.01) compared to the controls (Figure 3B).

Further statistical analysis showed that in the NEC-surgical group there were significant decreases in relative abundances of *Coprococcus* compared to the NEC-medical group and *Intestinibacter* and *Subdoligranulum* compared to the controls (*p* < 0.05). Furthermore, there was a significant increase in relative abundance of *Sutterella* in the NEC-surgical group compared to the control group (*p* < 0.05) (Figure 3B).

Consistent with these findings, within the NEC-surgical group, there were trends of decrease in relative abundance of *Romboutsia* and increase in relative abundance of *Corynebacterium* compared to the controls (0.1 > *p* > 0.05) (Figure S3B).

#### The relative abundance of microbial composition among the surgical and medical groups at species level

At species level, *Phocaeicola vulgatus* was among the most common in both the NEC-medical and the NEC-surgical groups, while there were dissimilar proportions between the groups regarding the other common bacteria (Figure 2F).

Our analysis unveiled strongly significant lower relative abundances of *Clostridium saccharogumia*, *Hydrogeniiclostidium mannosilyticum*, and *Haemophilus parainfluenzae* in NEC-surgical treated group versus control ones. In contrast, *Sutterella wadsworthensis* and *Bifidobacterium animalis* were significantly enriched in the NEC-surgical cases compared to the controls (*p* < 0.01) (Figure 3D).

Considering the impact of treatment on the microbiota composition of fecal samples from the NEC cases, the relative abundance of *Clostridium* sp. *AF15-31*, *Clostridium* sp. *TF06-15AC*, *Ellagibacter isourolithinifaciens*, *Faecalibacterium* sp. *OF04-11AC*, and *Staphylococcus aureus* were significantly lower in the NEC-surgical group than in the NEC-medical group (*p* < 0.05) (Figure 3D).

Our study indicated that there were trends of decrease of *Bifidobacterium catenulatum* in the NEC-surgical group compared to the controls (0.1 > *p* > 0.05) (Figure S3D).

#### Fecal microbiome diversity in all cases (treated [medical/surgical] NEC and control) at 5 years of age

**Figure 4:**
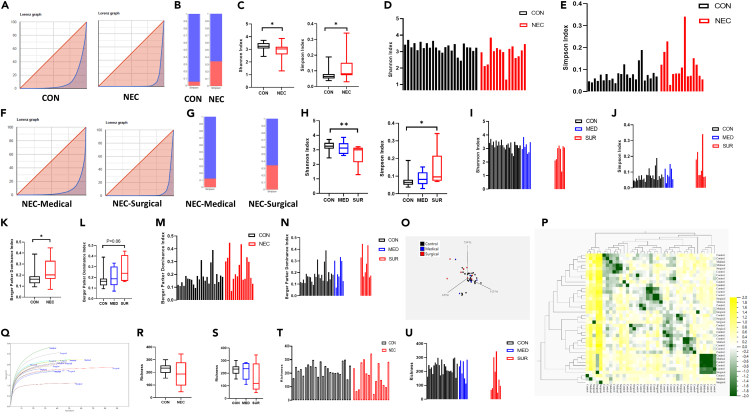
The diversity index across groups (A) Pink area in Lorenz graph took a larger percentage of the total area in NEC group, which was representative of a high degree of inequality. (B) Example of Simpson index in NEC and control cases. (C) Significant lower Shannon index and higher Simpson index in NEC group. (D) Distribution of Shannon index for each case within each group. (E) Distribution of Simpson index for each case within each group. (F) Pink area in Lorenz graph took a larger percentage of the total area in surgical NEC treated group, which was representative of a high degree of inequality. (G) Example of Simpson index in NEC-medical and NEC-surgical cases. (H) Significant lower Shannon index and higher Simpson index in NEC-surgical group compared to the control group. (I) Distribution of Shannon index for each case within each group. (J) Distribution of Simpson index for each case within each group. (K) Significant higher Berger Parker Dominance index in NEC group compared to the control group. (L) Trend of increase in Berger Parker Dominance index in NEC-surgical group compared to the control group. (M) Distribution of Berger Parker Dominance index for each case within each group. (N) Distribution of Simpson index for each case within each group by considering treatment. (O) Principal component analysis (PCA) analysis of the similarity of microbiota (OTUs) between the control, medical, and surgical groups. (P) Cluster heatmap of beta diversity included NEC and control groups with no significant different clustering. (Q) Rarefaction curve based on Shannon H index. (R and S) No difference in richness between the groups. (T and U) Distribution of richness for each case within each group. Data are presented as median, 1–99th percentile. Statistical comparison between control (*n* = 24) and NEC (*n* = 15) groups was performed using the non-parametric Mann-Whitney test and between the control (*n* = 24), NEC-medical (*n* = 7) and NEC-surgical (*n* = 8) groups was performed using the non-parametric and Kruskal-Wallis test. ∗*p* < 0.05; ∗∗*p* < 0.01. See also Figure S1.

Evaluating the NEC microbiome for differences in alpha-diversity indicated significantly lower Shannon index and higher Simpson index in the NEC group compared to controls (*p* < 0.05), suggesting that NEC-survivors had less species diversity and evenness (Figures 4A–4D). In the next step, the effect of treatment on alpha-diversity in the fecal microbiota was analyzed, showing significantly lower Shannon index in the surgically treated NEC group compared to the controls (*p* < 0.01), while Shannon index in the medically treated NEC group did not show a difference. Regarding the Simpson index, there was a statistically significant difference between the NEC-surgical group and the control group (*p* < 0.05) (Figures 4F–4J).

The Berger-Parker Dominance (BPD) Index was calculated to further evaluate alpha-diversity. The index measures the relative cover of the most abundant species regardless of species identity. The BPD index was significantly higher in NEC cases compared to the control group (*p* < 0.05). Regarding the effect of treatment on the BPD index, our results indicated a trend of increase in BPD index in the NEC-surgical group compared to the controls (Figures 4L–4O).

For beta-diversity, the results of Bray-Curtis dissimilarity matrix revealed that the overall composition of the NEC gut microbiota was not different from that of the control gut microbiota (Figures 4B and 4Q). The species richness analysis showed no significant differences among these three groups (Figures 4R–4V).

By performing spatiotemporal analysis, we found 2.31 ≤ Shannon index < 2.82 with the condition support (10.26%) and confidence (50%) in the NEC-medical group, and 1.80 ≤ Shannon index < 2.31 with the condition support (5.13%) and confidence (100%) in the NEC-surgical group. These results can be used as a prediction of dysbiosis based on the treatment. In the control group, Shannon index >3.34 with the condition support (28.21%) and confidence (81.82%) indicated the composition of gut microbiota. We also found that with the confidence of 100%, 36 ≤ number of days with antibiotics < 53 would predict 2.828 ≤ Shannon diversity index < 3.340.

## Discussion

This study set out to assess, for the first time, the metagenomic profiles of the intestinal bacterial population in preterm born children, looking at the effect of NEC on bacterial diversity 5 years after birth and, furthermore how much of this effect was mediated through NEC treatment (medical/surgical). We found that decreasing alpha-diversity and bacterial dysbiosis following NEC are likely to persist in children born preterm until they are 5 years old. More importantly, NEC treatment (medical/surgical) has differential long-term impact on microbiota composition of fecal samples from children born preterm.

Postnatal intestinal adaptation in premature infants is a critical process that involves various structural, functional, and immunological changes to support nutrient absorption, immune defense, and overall gut health.^27^ Prior studies have noted that the negative influence of several environmental and host conditions on the development of the gut microbiome in preterm infants is linked to necrotising enterocolitis (NEC).^28^ The incidence of NEC in preterm infants is about 13% in developed countries, with an increasing incidence rate over time.^29^ NEC may manifest pathologies such as inflammatory pathways with underlying microbiome dysbiosis.^30^

We found the notable difference in trend patterns observed between surgical and medical treated cases over the number of PN days, which suggests that there may be unique characteristics or circumstances associated with PN that contribute to the observed outcomes in the NEC-surgical group. Compared to full-term infants, the composition of human milk from mothers of preterm infants is higher in protein, fat, free amino acids, and sodium, which tend to decrease with time.^31^ Further investigation is warranted to uncover the underlying factors driving these trends.

Previous studies have explored links between differential patterns of intestinal microbiota in preterm and term infants with the mode of delivery, antibiotic exposure, and type of feeding.^32^ Consistent with the literature, our research indicated a significantly prolonged antibiotic therapy in the NEC group compared with the children without a history of NEC. It was applicable for both NEC-medical and NEC-surgical groups. Our results also indicated a significant negative correlation between the duration of antibiotic exposure and Shannon index and a significant positive correlation with Simpson index in all cases (with and without NEC history). However, other factors such as height, BMI, and weight at five years of age were not associated with dysbiosis. Animal models of NEC have also demonstrated that dysbiosis, characterized by an imbalance in the gut microbiota composition, is associated with NEC development similar to what is observed in human preterm infants with NEC.^33^ The clinical significance of the crosstalk between initial gut microbiota composition and health outcomes suggests that changes in the gut microbiota in preterm infants is related to poor neonatal growth and possibly to neurodevelopment disorders later in life.^34^ Our findings indicated less weight gain in surgically treated NEC children compared to children without a history of NEC. Therefore, our results suggest that the surgical treatment of NEC may affect the later gut microbiota composition, with the consequence of poor growth of children later in life. Future animal studies are needed to complement our clinical findings and provide further insights into disease mechanisms.

Our results showed less species diversity and evenness of the intestinal microbiota in 5-year-old children born preterm with a history of NEC during the neonatal period, compared to children without a history of NEC. The most important clinically relevant finding of our study was significantly impaired alpha-diversity in surgically treated NEC cases compared with the controls without NEC history. This finding is consistent with a study where preterm neonates with NEC had lower Shannon diversity scores than their healthy preterm counterparts.^35^ Moreover, we found that both Shannon and Simpson index were changed significantly in children with a history of NEC that underwent surgical treatment. This result may be explained by the fact that Shannon index is mostly affected by both predominant and rare bacteria, while Simpson index is mostly related to the prominent bacteria,^36^ indicating that dysbiosis in surgically treated NEC children comes from both predominant and rare bacteria alteration.

Full-term infants showed a relative abundance of 46% Proteobacteria and 35% Firmicutes at the age of one year,^37^ but adult samples had Firmicutes and Bacteroidetes phyla as the most common ones.^38^ Our results from five-year-old children born preterm with and without NEC history showed a relative abundance of 1–4% Proteobacteria and 58–62% Firmicutes, which indicated similarity in the predominant bacteria at phyla level with adult samples. This suggests the same pattern of intestinal microbiota signature at phyla level between term and preterm cases at the age of 5 years. However, considering the type of treatment in cases with a history of NEC indicated that the relative abundance of Proteobacteria was higher in surgically treated (6.36%) than in medically treated (2.27%). Consistent with our findings, several human studies also pointed an association between Gammaproteobacteria and increased risk for NEC in preterm infants.^35^ Moreover, preclinical studies have identified an overabundance of Gammaproteobacteria that activates TLR4 signaling has been linked to NEC development, highlighting the role of specific microbial taxa in NEC pathogenesis.^39^ Therefore, our findings suggest that colonization of gut microbiota in children with surgically treated NEC is more like early-life (one-year-old) intestinal microbiota than the older ones, which emphasizes immaturity in gut microbiota colonization in surgically treated NEC cases.

From our findings, it is likely that such a key difference in determining pathogenesis is the relative abundance of a single dominant species level, as we found increased abundance of *Sutterella wadsworthensis* (phylum Proteobacteria) in the stool of children with a history of NEC. Importantly, we observed that the type of NEC treatment had a significant long-term impact on *Escherichia coli* composition at species level, which was in the surgical group 18.21% while in the medical group was 6.33%. This suggests that not only the NEC pathology itself but also the treatment strategies play a crucial role in shaping the gut microbiota profile.

Based on our findings, relative abundance of *Akkermansia muciniphila* as a beneficial bacterium for suppressing inflammation and reducing oxidative stress^40^ was the least common species in surgical group; nevertheless, it was the most common bacteria in medical group.

This finding supports evidence from previous observations about increased relative abundance of bacteria from the Proteobacteria phylum in the stool of infants who develop NEC. However, it also indicated that maybe not the NEC pathology itself, rather treatment approach of NEC strongly influences the gut microbiota composition.

*Bacteroides caccae* was significantly decreased in the NEC group and specifically in the NEC-surgical group. *Bacteroides* has an anti-inflammatory function through surface component polysaccharide. Therefore, the decrease of the *Bacteroides caccae* may impact signaling pathways involved in critical gastrointestinal inflammatory function in preterm infants who developed NEC. These results are consistent with other research that found proinflammatory, low diversity gut microbiota composition in stool samples of preterm infants.^41^

It has been shown that preterm infants have delayed colonization with *Bifidobacteria*, leading to the proinflammatory status in the gut composition.^42^ One interesting finding is that the alteration in *Bifidobacteria* species does not follow the same pattern as we found a significant increase in relative abundance of *Bifidobacterium animalis* in children with surgically treated NEC compared with the children without NEC history, in contrast with a reduction in relative abundance of *Bifidobacterium angulatum* and *Bifidobacterium catenulatum*.

### Limitations of the study

The limitation of this study is the relatively small sample size that may affect the applicability of the results. However, all included cases in this study were carefully selected by having detailed information and follow up. A structured study protocol was followed for all included cases while collecting the samples. Another strength is the ability to collect stool samples at 5 years of age from 39 children born preterm at a single center.

## STAR★Methods

### Key resources table

**Table:** 

REAGENT or RESOURCE	SOURCE	IDENTIFIER
**Chemicals, peptides, and recombinant proteins**		

Shotgun metagenomics sequencing	Clinical Microbiomics; Copenhagen, Denmark	
NucleoSpin 96 Soil kit	Macherey-Nagel	740787.2
ZymoBIOMICS Microbial Community Standard	Zymo Research	Cat# D6300
NCBI RefSeq archaea, bacteria, fungal, protozoa, and viral genomes	NIH	https://www.ncbi.nlm.nih.gov/refseq/

**Software and algorithms**		

CheckM	Parks et al.^31^	https://ecogenomics.github.io/CheckM/
Bowtie2 (Version. 2.4.2)	Langmead et al.^32^	https://bowtie-bio.sourceforge.net/bowtie2/index.shtml
AdapterRemoval (Version. 2.3.1)	Schubert et al.^33^	https://github.com/MikkelSchubert/adapterremoval
BWA mem (Version. 0.7.17)	Li et al.^34^	https://bioweb.pasteur.fr/packages/pack@bwa@0.7.17
iNEXT	Chao, Hsieh, T.C. & Chao, A. et al.^37^^,^^38^^,^^39^	https://chao.shinyapps.io/iNEXTOnline/
Qlucore Software	Lund, Sweden	https://qlucore.com/
SPSS (IBM Corp. Version. 28.0)	Armonk, NY, USA	https://www.ibm.com/spss
GraphPad Prism 8	La Jolla, California, USA	https://www.graphpad.com/features

**Biological samples**		

Fecal samples of human	Queen Silvia Children’s Hospital in Gothenburg, Sweden	

### Resource availability

#### Lead contact

Further information and requests for resources should be directed to and will be fulfilled by the lead contact Anders Elfvin (anders.elfvin@vgregion.se).

#### Materials availability

This study did not generate new unique reagents.

#### Data and code availability

All data reported in this paper will be shared by the lead contact upon request.

Any additional information required to reanalyze the data reported in this paper is available from the lead contact upon reasonable request.

This paper does not report original code.

### Method details

#### Study participants and samples’ handling and collection

In this study, we analyzed fecal samples collected at 5 years of age, median age 5.1 years (4.3–6.0 years), from children born preterm with and without a history of NEC during the neonatal period. As the availability of cases for inclusion in the study was limited, which restricted the sample size, a power calculation was not performed. Criteria for inclusion was a minimum of stage IIa NEC according to the Bell’s staging criteria modified by Walsh and Kliegman.^43^ Both medically and surgically (surgery through the abdominal wall, i.e., laparoscopy or laparotomy) treated NEC were included. Detailed patient selection information can be found in supplementary and in a recent publication from our group.^44^ Figure 1 presents data regarding the inclusion of study subjects. In a previously published study regarding NEC, bone mass, and body composition, 25 children born preterm who developed NEC during their neonatal period, treated at Queen Silvia Children’s Hospital in Gothenburg, Sweden, were included at five years of age (27). For every NEC case, one control matched for gestational age, sex, and age at inclusion in the study but without a history of NEC, was initially included. Ten of the initially included 25 NEC cases, and one of the 25 controls failed to deliver fecal samples and were excluded from this study. The hospital’s medical records were used to collect data regarding birth, infant characteristics, events in the neonatal period, and surgical events. The medical records were also used to collect enteral and PN data. The Swedish Neonatal Quality Register (SNQ) was used to collect data on antibiotic use during their first admission in the NICU. SNQ and the medical records were used to find specific diagnoses, i.e., intraventricular hemorrhage (IVH), retinopathy of prematurity (ROP) and bronchopulmonary dysplasia (BPD). IVH is described in four grades, 1–4. This study defined severe IVH as a minimum of grade 3. PDA was defined as an open ductus arteriosus that required medical and/or surgical treatment. Surgical treatment was either a first choice or secondary after medical treatment with indomethacin. In some infants where surgery was required as treatment for NEC, the PDA surgery was done at the same surgical occasion. BPD was defined as a supplemental oxygen requirement at 36 weeks corrected gestational age, and severe BPD was defined as dependent of ≥30% oxygen and/or requirement of CPAP or ventilator at 36 weeks corrected gestational age. ROP is categorized into five severity stages, 1–5, where ≥ stage 3 was defined as severe ROP in this study.

#### Collection of fecal samples and analysis of microbiota

Fecal samples were collected once at 5 years at home using a fecal sample collection kit with a fecal spatula and a tube. Samples were brought to the hospital by the family on the same day as they were collected. They were initially frozen at −20°C and transported on ice, then frozen at −80°C and analyzed using the shotgun metagenomics sequencing method^26^ (Clinical Microbiomics; Copenhagen, Denmark).

#### DNA extraction and sequencing

DNA was extracted from ∼0.1 g aliquots of the fecal samples using the NucleoSpin 96 Soil (Macherey-Nagel) kit. A minimum of one negative control was included per batch of samples from the DNA extraction and throughout the laboratory process (including sequencing). A ZymoBIOMICS Microbial Community Standard (Zymo Research) was also included in the analysis as a positive (mock) control. Before library preparation, the DNA was quantified by Tecan Infinite F Nano+ Plate Reader using Quant-iT dsDNA BR Assay Kit. The genomic DNA was normalized to 10 ng/μL (100 ng input). The enzymatic fragmentation of DNA and library construction was conducted by Tecan DreamPrep NGS using Celero EZ DNA -seq Core Module Kit. The fragmented DNA was amplified using PCR. Short and large DNA fragments were removed using double-sided magnetic bead size selection (AMPure XP, Beckman Coulter). During library construction, adapter sequences from Celero 96-Plex Adaptor Plate were added to each sample. Tecan Infinite F Nano+ Plate Reader using NuQuant NGS Library Quantification Module and Qubit quantified the final concentration for each library. The final fragment distribution is evaluated using a Fragment Analyzer 5200 (Agilent). Qubit and TapeStation were used to determine the concentration of the final library before sequencing. The library was sequenced using 2 × 150 bp paired-end sequencing on an Illumina platform.

#### Gene catalog and MGS definitions

As a reference gene catalog, we used the Clinical Microbiomics Human Gut HG04 gene catalog (14,355,839 genes), which was created based on 12 170 non-public deep-sequenced human gut specimens (including 481 from infants), 9428 publicly available metagenomes compiled from 43 countries^45^ and 3567 publicly available genome assemblies from isolated microbial strains. For taxonomic abundance profiling, we used the Clinical Microbiomics HGMGS version HG4.D.2 set of 2095 metagenomic species (MGS), each represented by a set of genes with highly coherent abundance profiles and base compositions in the 12 170 metagenomes. The metagenomic species concept is described in Nielsen et al.^26^ To taxonomically annotate an MGS, we blasted its genes against NCBI RefSeq archaea, bacteria, fungal, protozoa, and viral genomes (2022-01-19) and nt (2021-08-03) databases and used rank-specific annotation criteria. Specifically, we assigned a taxon to an MGS if at least M % of its genes were mapped to the taxon and no more than D % of its genes were mapped to a different taxon. We only considered blast hits with an alignment length ≥100 bp, ≥50% query coverage and % identity ≥ PID. Here we define: PID = (95, 95, 85, 75, 65, 55, 50, 45); M = (75, 75, 60, 50, 40, 30, 25, 20); and D = (10, 10, 10, 20, 20, 20, 20, 15) for subspecies, species, genus, family, order, class, phylum, and superkingdom, respectively. Finally, we processed each MGS with CheckM^46^ and updated our annotation with the CheckM result if this resulted in a lower taxonomic rank.

#### Sequencing data preprocessing

Raw FASTQ files were filtered to remove host contamination by discarding read pairs in which either read mapped to the human reference genome GRCh38 with Bowtie2 (v. 2.4.2),.^47^ Reads were then trimmed to remove adapters and bases with a Phred score below 30 using AdapterRemoval (v. 2.3.1),.^48^ Read pairs in which both reads passed filtering with a length of at least 100 bp were retained; these were classified as HQNH reads.

#### Mapping reads to the gene catalog

HQNH reads were mapped to the gene catalog using BWA mem (v. 0.7.17).^49^ An individual read was considered uniquely mapped to a gene if the mapping quality (MAPQ) was ≥20 and the read aligned with ≥95% identity over ≥100 bp. However, if > 10 bases of the read did not align with the gene or extend beyond the gene, the read was considered unmapped. Reads meeting the alignment length and identity criteria but not the MAPQ threshold were considered multi-mapped. Each read pair was counted as either 1) uniquely mapped to a specific gene, if one or both individual reads were uniquely mapped to a gene, or 2) multi-mapped if neither read was uniquely mapped, and at least one was multi-mapped, or 3) unmapped if both individual reads were unmapped. If the two reads were each uniquely mapped to a different gene, the gene mapped by read 1 was counted but not the gene mapped by read 2. A gene count table was created with the number of uniquely mapped read pairs for each gene. Details are available in supplementary.

#### MGS relative abundance calculation

For each MGS, a signature gene set was defined as the 100 genes optimized for accurate abundance profiling of the MGS. An MGS count table was created by counting the number of reads uniquely mapped to the MGS signature genes per sample. An MGS was considered detected if reads from a sample uniquely mapped to at least three of its signature genes; measurements that did not satisfy this criterion were set to zero. Based on internal benchmarks, this threshold results in 99.6% specificity. The MGS count table was normalized according to effective gene length and then normalized sample-wise to sum to 100%, resulting in relative abundance estimates for each MGS. Down sampled (rarefied) MGS abundance profiles were calculated by random sampling, without replacement, of a fixed number of signature gene counts per sample, and then following the procedure described above. In this study, 524050 signature gene counts were sampled.

#### Diversity estimates

Alpha and beta diversity estimates were obtained from rarefied abundance matrices created by random sampling of reads without replacement. Alpha diversity of the fecal microbiota was assessed with Shannon diversity index, Simpson index, richness, and BPD index which were obtained from the number of OTUs assigned at the species level as described before.^50^ BPD index is calculated due to its mathematical independence from richness.^51^ Bray-Curtis dissimilarity matrix for beta diversity was calculated.

#### Bioinformatic and statistical analysis

All sequencing data were computed using the R-based interactive of iNEXT software, based on statistical estimation of the true Hill number of any order q ≥ 0 for diversity estimation.^52^^,^^53^^,^^54^ Heatmap and PCA were generated by applying Qlucore Software (Lund, Sweden). Lorenz curve is generated to represent inequality in the distribution of fecal microbiota (alpha diversity). Statistical analysis was performed using SPSS (IBM Corp. Version 28.0. Armonk, NY, USA). Graphs were created using Prism 8 (GraphPad Software Inc., USA) and Excel (Microsoft, Excel, 2019). Comparison of data between control and NEC subjects and between control, NEC-medical treatment, and NEC-surgical treatment subjects was conducted using Mann–Whitney U test and Kruskal–Wallis test, respectively. Correlation analysis between the parameters was done using Spearman correlation test. The Hedges' g score was calculated to test the effect size of delivery type (normal vaginal/cesarean section) was on the alpha diversity index. Significance values have been adjusted by the Bonferroni correction for multiple tests. Spatial-temporal association modeling was used to predict the alpha diversity index based on antibiotic therapy and treatment duration as conditions by indicating percentage of condition support and confidence.^55^^,^^56^

#### Ethics

The study was approved by the Regional Ethical Review Board of Gothenburg, Sweden (720-13 and 319-12), with an amendment approved by the Swedish Ethical Review Authority Dnr 2022-03402-02. Written informed consent was obtained from all parents or caregivers.

## Footnotes

Supplemental information can be found online at https://doi.org/10.1016/j.isci.2024.110325.
