# Supplementary material for: Gut microbiota differences in five-year-old children that were born preterm with a history of necrotizing enterocolitis: A pilot trial
Source: iScience. 2024 Jun 20;27(7):110325. doi: 10.1016/j.isci.2024.110325 (PMC11269947; doi:10.1016/j.isci.2024.110325)
Supplement: Document S1. Figures S1–S3 and Table S1 [file mmc1.pdf]

iScience, Volume *27*

Supplemental information

Gut microbiota differences in five-year-old children that were born preterm with a history of necrotizing enterocolitis: A pilot trial

Amanda Magnusson, Seyedeh Marziyeh Jabbari Shiadeh, Maryam Ardalan, Diana Swolin-Eide, and Anders Elfvin

Supplementary table 1: Sequencing read quality control and gene catalog mapping statistics. Average, minimum and maximum values of quality control and read mapping statistics, related to Figure 1.

	Min	*Average*	*Max*
Read pairs	14,6 M	40,3 M	716,6 M
High quality reads	14,5 M	40,1 M	712,6 M
High quality non-host reads	13,2 M	40,1 M	712,2 M
Mapped to gene catalog	10,8 M	32,9 M	580,2 M
Gene catalog representation	78,2 %	82,8 %	85,7 %

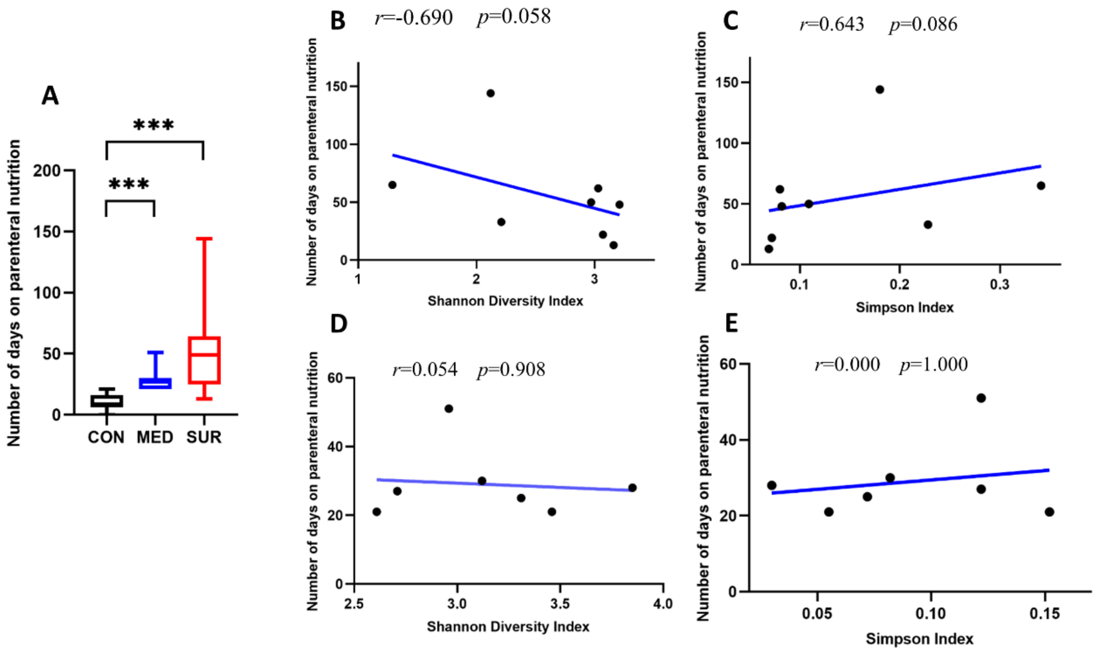

Figure S1: Number of days on parenteral nutrition and dysbiosis. Comparison of number of days on parenteral nutrition between control, NEC-medical and NEC-surgical treatment groups. ***$p < 0.001$ (A). Negative correlation between number of days on parenteral nutrition and Shannon index in surgical-treated group (B). Positive correlation between number of days on parenteral nutrition and Simpson index in surgical-treated group (C). Non-significant correlation between number of days on parenteral nutrition and Shannon index in medical-treated group (D). Non-significant correlation between number of days on parenteral nutrition and Simpson index in medical-treated group (E), related to Figure 4.

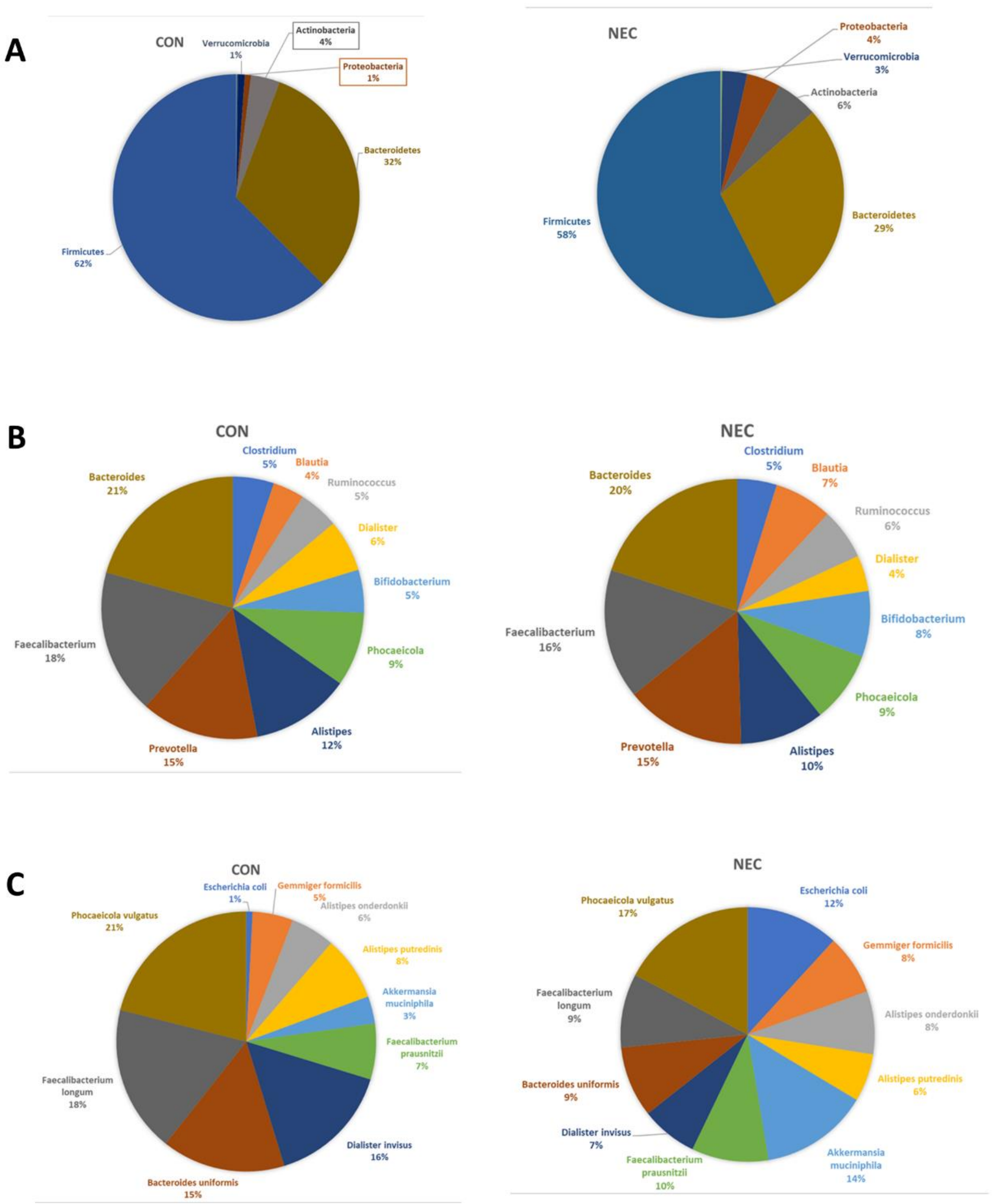

Figure S2: Percentage of most common bacteria. Pie charts display the percentage of the most common bacteria at the level of phylum (A), genus (B) and species (C) in control and NEC groups separately, related to Figure 2.

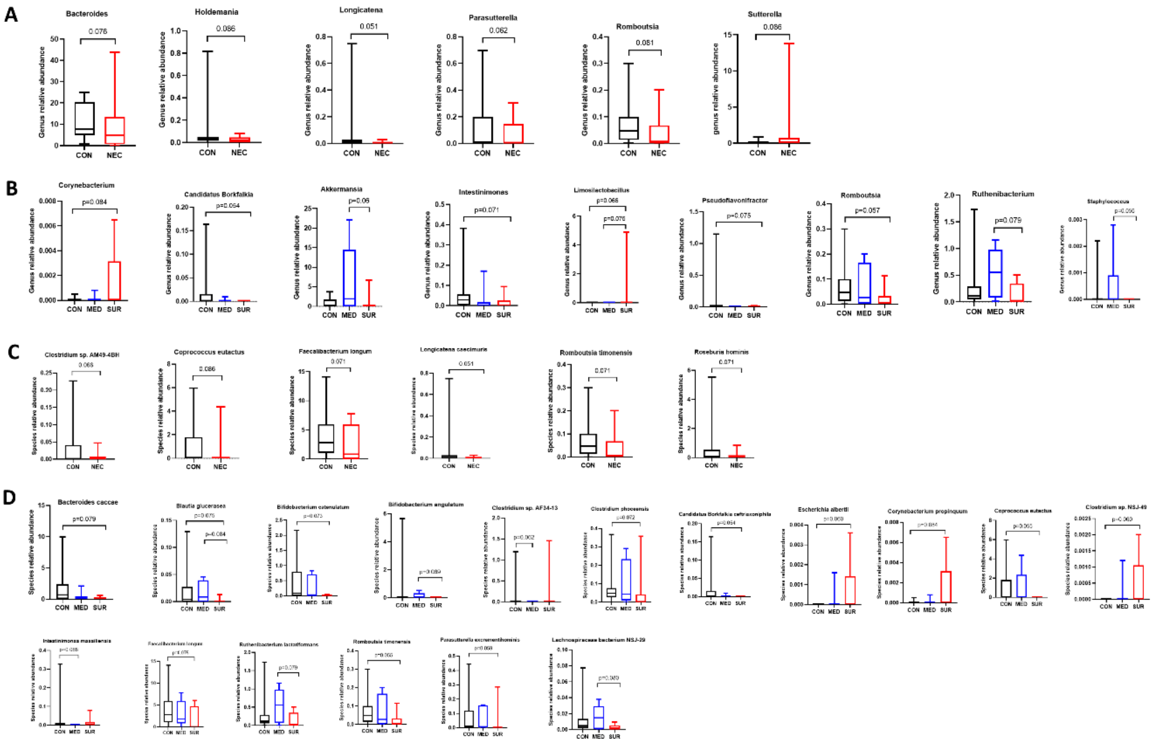

Figure S3: Trend in difference in the relative abundances of taxa at the genus level between groups. Comparison of the relative abundances of taxa at the genus level between NEC and control participants by considering trend in difference between two groups (A). Comparison of the relative abundances of taxa at the genus level between control, NEC-medical and NEC-surgical treatment groups by considering trend in difference between the groups (B). Comparison of the relative abundances of taxa at the species level between NEC and control participants by considering trend in difference between two groups (C). Comparison of the relative abundances of taxa at the species level between control, NEC-medical and NEC-surgical treatment groups by considering trend in difference between the groups (D), related to Figure 3.
